# Supplementary material for: Assessing the healthiness of UK food companies’ product portfolios using food sales and nutrient composition data
Source: PLoS One. 2021 Aug 4;16(8):e0254833. doi: 10.1371/journal.pone.0254833 (PMC8336824; doi:10.1371/journal.pone.0254833)
Supplement: Attachment — Submitted filename: ETsReview.docx [file pone.0254833.s001.docx]

ET’s review:

Thank you for the opportunity to review this manuscript – assessing the healthiness of UK food companies’ product portfolios using food sales and nutrient composition data. Overall, the manuscript is written well, and the methods applied are thorough. I have a number of comments that could further improve the manuscript.

Abstract
- in methods, the threshold of nutrient profile score for classifying products as healthy vs. unhealthy hasn’t been provided. Describing briefly the threshold will help readers understand better the results.

- Line 48, is it ‘only 6 brands among the 10 companies improved …’ ?
- Lines 46 and 47 ‘… the number of products classified as healthy (46% to 48%)…’ the statement says ‘the number’ but results are in ‘%’
- Line 49, proportion of total volume sales (classed?) classified as healthier increased from 44% to 51% …; but in line 52, the proportion of foods classified as healthy decreased from 7% to 6%... is confusing. It’s important to be consistent which means the decrease should be the ‘proportion of total volume sales classified as healthier’. Moreover, the use of healthier in some places and healthy in other places is confusing. The authors may choose to use ‘healthy’ whenever ‘healthier’ refers to ‘healthy’ as a binary variable created using the threshold of ≥62 for foods and ≥66 nutrient profiling score for drinks.

Methods

- Line 108, ‘… data were automatically matched based on product name, brand name, category and year, …’ needs to be explained on how the authors created the identifier variable in the sales database and nutrient composition database. By identifier variable I mean the variable that was used to link (match) the two databases.
- Line 111, ‘… The matching code was adjusted, …’ The authors please explain how this adjustment was done.
- Line 142, ‘… weighted mean nutrient profiling scores were calculated for each company and brand, …’. It is well written here; however, in figure3 and other figures it says ‘sales-weighted score’. The authors may choose to clarity it here or may choose to change ‘Variable calculated’ to ‘Sales-weighted score’ in line 140.
- Line 146, … Infant formulas (i.e., breast milk substitutes) were excluded. Were there other products to be excluded such as products not required to display a Nutrition Information Panel (e.g. tea, unflavored coffee, artificial sweeteners, chewing and bubble gums, salt, flour, corn flour, vinegar, herbs and spices, pepper, baking soda, baking powder, tartaric acid, citric acid, cooking ingredients, ice, curry powder, yeast, bicarbonate of soda. Other foods such as special products (baby foods, protein bars, protein powders, and fitness or diet products), and alcoholic beverages may also be excluded. If these products were not available in the database, then the authors may mention about them.

Results

- In Table 1. What does ‘per capita per day’ refer to?
- Line 173, does ‘healthier’ mean ‘healthy’? based on the threshold described in line 125
- Line 173 says ‘… increase from 44% to 51% …’ but in line 174 after removing soft drinks from the analysis, the proportion of volume sales classified as healthier decreased from 7% to 6% (p=0.33). I assume that 7% refers to 51% minus 44%, and 6% refers to 50% minus 44%. I am not sure about it, and it think its needs to be clarified here.
- I am unsure whether ‘bottled water’ should be excluded from the analysis, especially it may favor manufacturing of more healthy beverages by Coca-Cola, and PepsiCo.
- Results in page 8 showing significant improvement in nutrient profiling score for soft drink brands give the impression that these brands (i.e., San Pellegrino, Fanta, Volvic, Tropicana, Kellogg) are doing good in manufacturing more healthy beverages. I’m not sure whether inclusion of ‘bottled water’ in the analysis has affected the results or whether reductions in salt, sugar and energy have led to these results.

Discussion

- Line 238 ‘… foods and drinks available, I understand the authors studied sales; but did the authors examine availability too?
- Line 240 ‘… responding to public health calls to improve …’ is not specific. The authors may choose to write ‘… responding to the voluntary reformulation initiatives to improve’
- Lines 246 to 274 mainly describe and discuss ‘missing values’ and ‘imputation’. They can be divided between methods, results and discussion. If the authors think the results related to missing values, imputation and sensitivity analysis can distract the readers, the authors may choose to provide them as an appendix.
- Line 261, … (-0.5%) refers to what?
- Line 288, please cite the references in ‘… energy dense foods in Mexico [reference?] and mandatory warning labels in Chile [reference?]’.

One of the strengths of this study can be use of nutrient profiling score that the authors may want to highlight it in the discussion. In this regard lines 312 and 313 say … these analyses consider only single nutrients and do not apply a nutrient profile model …’ but these are not emphasizing on this strength of this study.
